# The Roles of the NLRP3 Inflammasome in Neurodegenerative and Metabolic Diseases and in Relevant Advanced Therapeutic Interventions

**DOI:** 10.3390/genes11020131

**Published:** 2020-01-27

**Authors:** Rameez Hassan Pirzada, Nasir Javaid, Sangdun Choi

**Affiliations:** Department of Molecular Science and Technology, Ajou University, Suwon 16499, Korea; rameez_hassan99@yahoo.com (R.H.P.); nasirjavaid1989@gmail.com (N.J.)

**Keywords:** inflammasome, Alzheimer’s disease, type 2 diabetes mellitus, machine learning, artificial intelligence

## Abstract

Inflammasomes are intracellular multiprotein complexes in the cytoplasm that regulate inflammation activation in the innate immune system in response to pathogens and to host self-derived molecules. Recent advances greatly improved our understanding of the activation of nucleotide-binding oligomerization domain-like receptor (NLR) family pyrin domain containing 3 (NLRP3) inflammasomes at the molecular level. The NLRP3 belongs to the subfamily of NLRP which activates caspase 1, thus causing the production of proinflammatory cytokines (interleukin 1β and interleukin 18) and pyroptosis. This inflammasome is involved in multiple neurodegenerative and metabolic disorders including Alzheimer’s disease, multiple sclerosis, type 2 diabetes mellitus, and gout. Therefore, therapeutic targeting to the NLRP3 inflammasome complex is a promising way to treat these diseases. Recent research advances paved the way toward drug research and development using a variety of machine learning-based and artificial intelligence-based approaches. These state-of-the-art approaches will lead to the discovery of better drugs after the training of such a system.

## 1. Introduction

The human body has the ability to combat a pathogenic attack with the help of two kinds of immune system, i.e., the innate and the adaptive immune systems. There are many markers of the activation of these immune systems; one of them is inflammation. The latter is an evolutionary protective immune response that is tightly controlled by the innate immune system—against pathogens, cellular debris, and harmful stimuli. The innate immune system plays an essential role in the sensing of invading pathogens and of endogenous damage signals [1]. Dysregulation of inflammatory pathways can cause insufficient or excessive inflammation that either causes persistent infection or leads to systematic inflammatory diseases, respectively. 

Inflammasomes are multiprotein complexes with an intrinsic ability to initiate an innate immune response upon the recognition of a pathogen-associated molecular pattern (PAMP) or a damage-associated molecular pattern (DAMP). These molecular patterns are recognized by specialized structures, called pattern recognition receptors (PRRs), in the cytoplasm (e.g., RIG-I-like receptors: RLRs), on the cell surface, or in endosomal compartments (e.g., Toll-like receptors: TLRs) [2]. Engagement of these PRRs triggers downstream signaling pathways that lead to the production of proinflammatory cytokines [1,3]. Some of these cytokines are produced in their precursor form, which needs to be matured in order to become functionally active. This maturation requires the action of other key cellular players such as inflammasomes, which eventually cause the secretion of active cytokines from the cell as inflammatory markers. Inflammasome activation is mediated by the innate immune system; the underlying mechanism was explored recently in various studies [4,5,6].

The major components of the inflammasome complex are PRRs, including nucleotide-binding oligomerization domain (NOD)-like receptors (NLRs) and “absent in melanoma 2-like receptors” (ALRs, AIM2-like receptors) in both humans and mice [7]. So far, several inflammasomes have been identified, which include NLR family pyrin domain containing 3 (NLRP3), NLRP1, AIM2, and NLRC4 types. The NLRP3 belongs to the subfamily of NLRP with pyrin domain (PYD) at their N-terminal which is studied thoroughly due to its critical role in inflammatory and immune system-related disorders [8,9,10]. Besides, it contributes to the pathogenesis of a variety of neurodegenerative diseases (multiple sclerosis, Parkinson’s disease, and Alzheimer’s disease [AD]) and metabolic diseases (obesity, type 2 diabetes mellitus [T2D], and atherosclerosis) [11,12]. Moreover, the genetic polymorphisms and mutations in NLR-coding genes and in inflammasome sensor proteins are associated with a variety of autoimmune diseases [13,14]. This association with various diseases has led to the development of therapeutics that target inflammasome activity. The complexity of the biological system has paved the way toward cutting edge machine learning (ML) approaches in the field of discovery and development of drugs with enhanced therapeutic efficacy [15]. In this regard, virtual screening (VS) has played a critical part because it facilitates in silico screening of millions of compounds, and the latter process results in the identification of potential drugs. ML is a subset of artificial intelligence (AI) methods and is emerging as a powerful technique for VS, which compiles and trains a dataset (compounds) to classify it into known “actives” and “inactives.” The accuracy of the trained model is validated by its testing on raw datasets to characterize novel compounds with desired pharmacological properties [15,16].

The focus of this review is on our recent elucidation of NLRP3’s mechanism of activation and its participation in the pathogenesis of obesity/T2D and AD. Furthermore, we will discuss the ability of ML and AI to improve the discovery of new therapeutic approaches.

## 2. The NLRP3 Inflammasome

NLRP3 was initially characterized in an autoinflammatory disease named Muckle–Wells syndrome [17]. The NLRP3 inflammasome complex is mainly composed of three units: a receptor protein (NLRP3), an adaptor protein (ASC), and an effector protein (caspase 1) [18,19]. The receptor protein acts as a sensor that is switched on after sensing a PAMP and/or DAMP. The ASC adaptor protein contains two death domains: The N-terminal pyrin domain (PYD) and the C-terminal caspase recruitment domain (CARD) [19,20], which serves as a mediator between the sensor and effector protein.

The NLRP3 complex is primarily expressed in immune cells such as inflammatory and antigen-presenting cells (APCs). It is comprised of central nucleotide-binding and oligomerization domain (NBD), a C-terminal leucine-rich repeat LRR domain, and an N-terminal pyrin domain. NLRP3 as a receptor protein is stimulated by the presence of PAMPs and/or DAMPs including nucleic acids, lipooligosaccharide (LPS) and muramyl dipeptide (MDP) [21,22]. NLRP3 activation is divided into two steps: priming and activation [23]. The priming signal (first signal) includes a large number of DAMPs and/or PAMPs which activate PRR (such as TLR) signaling to stimulate the nuclear factor-κB (NFκB) pathway. This pathway promotes the transcription and expression of NLRP3 along with pro-IL-1β and pro-IL-18 that are translocated from the nucleus to cytoplasm in an inactive form [24]. Moreover, the priming signal includes injury-related factors such as oxidized low-density lipoprotein [25], whose accumulation has been identified in metabolically induced obesity-related diseases. The activating signal (second signal)—that initiates NLRP3 inflammasome stimulation—originates from a variety of activators such as PAMPs, DAMPs, exogenous adenosine, amyloid β (Aβ), mitochondrial DNA, or substances (e.g., asbestos and aluminum or silica and uric acid crystals) [26,27,28]. Furthermore, ATP-induced P2X7R activation raises K^+^ efflux, which is also critical for NLRP3 activation [29]. Eventually, ASC via the CARD domain mediates the recruitment of pro-caspase 1, which drives NLRP3 inflammasome assembly. The proximity to neighboring pro-caspase 1 leads to its autocleavage and conversion into mature caspase 1, which subsequently cleaves the precursor cytokines (pro-IL-1β and pro-IL-18) into their mature forms (IL-1β and IL-18) [3,30]. This event next initiates pyroptosis, an inflammatory type of programmed cell death that is controlled by gasdermin D (GSDMD) [31]. In pyroptosis, pores are formed in the plasma membrane as the N-terminal fragment of GSDMD detaches from its C-terminal inhibitory domain and attaches to phosphoinositides, which oligomerize to produce membrane pores [32,33]. The formation of membrane pores disturbs the cellular osmotic potential, thereby leading to cell swelling and lysis with the ultimate release of mature IL-1β and IL-18 into the extracellular environment [31].

**Figure 1:**
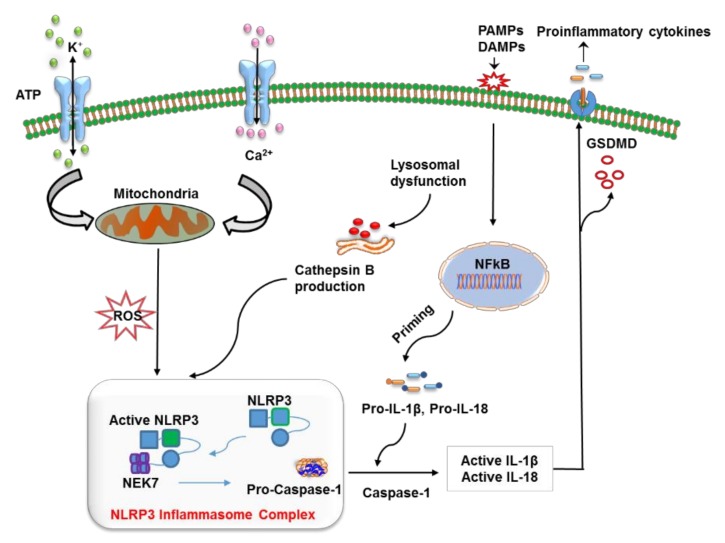
The mechanism of action of the nucleotide-binding oligomerization domain-like receptor (NLR) family pyrin domain containing 3 (NLRP3) inflammasome. The priming step involves the recognition of a pathogen-associated molecular pattern (PAMP) or a damage-associated molecular pattern (DAMP) by a specific pattern recognition receptors (PRR), which activates the NF-κB pathway to release precursor forms of IL-1β and IL-18 into the cytoplasm. NLRP3 is turned on by lysosome-mediated cathepsin B, K^+^ efflux, reactive oxygen species (ROS) production via dysfunctional mitochondria, the release of mitochondrial DNA in oxidized form, and alterations in Ca^2+^ concentration. The oligomerization and activation of NLRP3 take place after it interacts with the leucine-rich repeat (LRR) domain of NEK7. This event is followed by the cleavage of pro-caspase 1 into caspase 1, which converts pro-IL-1β and pro-IL-18 into their respective mature forms, which are finally released from the cell via pores generated by gasdermin D (GSDMD) (N-terminal fragments).

So far, numerous factors have been found to initiate NLRP3 inflammasome activation; however, the exact mechanism remains unclear and represents an area of active investigation. A possible mechanism underlying the initiation of the NLRP3 inflammasome includes reactive oxygen species (ROS) production and mitochondrial dysfunction, oxidized mitochondrial DNA release, K^+^ efflux, a cathepsin B release from disrupted lysosomes, changes in extracellular Ca^2+^ ion gradients, and the formation of transmembrane pores [34,35,36,37,38,39]. Moreover, NIMA-related kinase 7 (NEK7) has been reported to bind to the LRR domain of NLRP3 thus leading to its activation and oligomerization (Figure 1) [40]. On the other hand, opposing evidence highlights the controversial role of mitochondrial ROS in the inhibition of NLRP3 activation [41]. Moreover, studies have revealed that post-translational modifications are significantly involved in NLRP3 activation [3,42,43]. Nonetheless, ubiquitination and post-translational modifications at the priming step have been found to induce NLRP3 inflammasome inactivation [44,45], whereas dephosphorylation and deubiquitination cause its activation [43,46]. Additionally, protein kinase A–associated NLRP3 phosphorylation on residue Ser291 is important for NLRP3 inflammasome inactivation [47]. Moreover, recent studies uncovered the function of microRNAs in the regulation of the NLRP3 inflammasome. For example, myeloid-derived miR-223 controls intestinal inflammation by repressing the NLRP3 inflammasome [48]. miR-33 has also been demonstrated to regulate the NLRP3 inflammasome in macrophages because this pathway plays a critical part in the development of rheumatoid arthritis [49]. This observation provides further insights into the events that epigenetic regulators induce in this inflammasome.

Thus, numerous stimuli are recognized by their receptors and serve as first, second, or both signals to make NLRP3 active; nevertheless, further research is necessary to determine the exact mechanism.

## 3. Roles of NLRP3 Inflammasomes in Metabolic and Neurodegenerative Diseases

NLRP3 was initially characterized as a major causative factor of inflammation owing to NLRP3’ involvement in a group of rare heterogeneous autoinflammatory conditions, known as cryopyrin-associated periodic syndrome [50]. On the other hand, its activation by damage-associated stimuli activates innate response to tissue damage. Therefore, inflammasomes significantly contribute to the pathogenesis of various diseases including dementia, multiple sclerosis, cancer, and gout [51,52,53]. The most common form of dementia (60–80%) is Alzheimer’s disease (AD) which leads to neurodegeneration by various mechanisms such as mitochondrial dysfunction, oxidative stress, and inflammation. Metabolic disorders such as obesity and type 2 diabetes (T2D) have a strong correlation to increased risk of AD [54,55,56]. This association involves increased accumulation of amyloid-β (Aβ) which is confirmed in various mouse models [57,58,59]. Obesity, T2D, and AD are affected by the involvement of NLRP3; here, we focus on their association with each other from a therapeutic point of view. 

### 3.1. Obesity and T2D

Anomalous activation of the innate immune system significantly contributes to the pathogenesis of metabolic disorders such as T2D [60,61]. The latter is a chronic condition that was formerly termed as adult-onset diabetes, which is characterized by hyperglycemia and—according to more recent insights—by relative insulin deficiency triggered by pancreatic β-cell dysfunction [62]. In addition to other factors, obesity is a major factor responsible for T2D worldwide. Its prevalence may further increase globally, with the highest projected prevalence rates in developing or low-income countries [63]. Therefore, deeper insights into obesity pathogenesis as a significant risk factor for T2D hold huge promise for obesity prevention and treatment [64,65].

Obesity has multifactorial pathogenesis, which includes the growth of adipocytes and increased infiltration of macrophages into adipose tissue (AT) thereby activating inflammatory pathways and causing chronic inflammation [66]. Obesity-induced alterations in adipocytes and macrophages cause insulin resistance (IR) with subsequent induction of AT fibrosis [67]. Besides energy conservation, ATs secrete adipokines, molecules that contribute (via endocrine, autocrine, and paracrine signaling mechanisms) to various physiological and pathophysiological conditions, thereby regulating inflammation and immunity, insulin sensitivity, and food intake [68]. Furthermore, a reduction in the secretion of insulin-sensitive adipokines accompanied by oversecretion of proinflammatory cytokines (TNF-α, IL-1β, and IL-6) is closely related to the etiology of various metabolic conditions. Other studies also point to the participation of NLRP3 in obesity because of its overexpression in AT [11,69,70,71,72,73]. Moreover, the results of studies on mouse models of obesity are consistent with this observation [74,75]. The release of proinflammatory cytokines (IL-1β and IL-18) is the main culprit behind the AT inflammation in obese subjects [76] and thus this contributes to the development of T2D. The detailed function of the NLRP3 inflammasome in the pathogenesis of T2D is explained in a systematic review [77].

The activation of innate-immunity cells (such as neutrophils and macrophages) owing to the emergence of a chronic proinflammatory state has been closely associated with IR [78,79]. Furthermore, the activation of inflammasomes is due to the priming signals produced by the engagement of PRRs such as TLRs [61,80,81]. These events contribute to the development of IR and liver fat storage in mice with diet-induced obesity because of macrophage infiltration into AT [82,83,84]. Additionally, overexpression of NLRP3 inflammasome components in AT is associated with the pathogenesis of obesity and therefore is directly associated with T2D, atherosclerosis, and myocardial infarction [11,85]. Increased amyloid polypeptide deposition in pancreatic islets is one of the key inducers of NLRP3 activation [86,87] via lysosomal disruption and high ROS concentrations in pancreas-infiltrating macrophages [88]. Additionally, in comparison to wild-type mice, NLRP3 knockout mice show substantial improvement in insulin sensitivity during a high-fat diet; this approach provides protection against obesity [11,89,90]. Activation of NLRP3 causes oversecretion of inflammatory cytokines such as IL-1β, which impair islet cell function and induce dysregulation of blood glucose levels, thus resulting in the development of T2D [91,92]. The surface expression of functional IL-1β receptors on pancreatic β cells and infiltrating macrophages further enhances the production of IL-1β and the diffusion of inflammatory signals through the NF-kB pathway, which might ultimately cause β-cell dysfunction [91,93]. Even the early stage of T2D is characterized by dysfunctional β cells and a decrease in insulin production.

The mechanism of IL-1β–induced IR is the reduced tyrosine phosphorylation of insulin receptor substrate 1 (IRS1) along with its mRNA expression; this factor significantly contributes to the regulation of blood glucose, thus blocking the downstream insulin signaling pathway; these changes lead to IR [94,95]. Additionally, IL-1β-induced expression of TNF-α promotes IR [95,96]. Abundance of IL-1β and IL-18 in AT stimulates CD8^+^ T cells thus contributing to the inflammation of AT and to IR by activation and recruitment of macrophages [97,98]. High-fat diet-induced IR in a mouse model can be reversed in the absence of IL1R1 because this approach relieves AT inflammation despite the presence of immune-cell infiltration [90,99]. Similarly, mice deficient in IL-1α are protected against obesity-induced glucose intolerance; therefore, the blockade of IL1R1 and IL-1α could be used in the treatment of IR and obesity. In this context, T2D can be treated through IL-1 antagonism, given that IL-1β has a potential to worsen the function of pancreatic β cells. On the contrary, at low levels, IL-1β activates β-cell proliferation and insulin production, which synergistically stimulate glucose disposal and inflammation [100]. These data confirm the participation of inflammatory mediators in pathological and normal metabolism. The exact mechanism of NLRP3 inflammasome activation in T2D is not completely understood; however, mitochondria have been identified as a key player in this phenomenon [101]. In obese and T2D patients, the substances secreted by dysfunctional mitochondria into the cytoplasm often serve as DAMPs, which activate the NLRP3 inflammasome. Moreover, a diet rich in fats and glucose induces the production of mitochondrial ROS owing to Miro1-induced effects on mitochondria in pancreatic β cells; these effects consequently cause an NLRP3-induced inflammatory response, thereby impairing insulin production [102,103]. Furthermore, thioredoxin (TRX)-interacting protein (TXNIP) disassociation causes the activation of NLRP3 inflammasomes [104,105]. Hence, therapeutics targeting the NLRP3 inflammasome could be employed in the treatment of T2D and obesity because considerable numbers of studies have shown the effectiveness of this strategy. The antagonists (for the associated signaling pathways) that show a good potential for the treatment of symptoms associated with T2D and obesity are shown in Table 1. Nonetheless, the detailed mechanism of inflammasome activation that leads to obesity and causes T2D as well as treatment modalities based on NLRP3 inhibition remains to be explored. In addition, the characterization of specific molecular pathways of immune–metabolic interactions (that induce organ dysfunction due to the damage caused by chronic inflammation) may also give us a new insight into the treatment of obesity and T2D. Thus, in light of various studies, a significant link has been identified connecting NLRP3 with obesity and T2D; therefore, targeting NLRP3 could be beneficial for the treatment of the above-mentioned metabolic diseases.

### 3.2. Alzheimer’s Disease

AD is the most common neurodegenerative disorder. It is clinically characterized by dementia and progressive cognitive impairment, which are caused by two main pathologies: Aβ plaques and neurofibrillary tangles [119]. The estimated yearly incidence and prevalence of AD rise significantly with age. The incidence rate varies among different age groups: among people aged between 65 and 69, the approximate incidence is 0.4%; in people aged over 90, this rate is nearly 10% [120]. A significant contributing factor to the pathogenesis of AD includes aggregation of Aβ into neurotoxic plaques [121]. Accumulation of Aβ launches several cellular responses mediated by microglia, a type of neuroimmune cell located throughout the brain [122]. Recent studies revealed that Aβ can initiate NLRP3 inflammasome signaling in microglia [123], which causes the production of proinflammatory cytokines and consequently induces inflammation [124]. Studies have uncovered the involvement of several inflammatory components including prostaglandins, chemokines, and cytokines that are expressed in postmortem brain tissues isolated from patients with neurodegenerative disorders including AD; moreover, their elevated expression has been identified in cerebrospinal fluid [125,126]. Astrocytes and microglia are the mediators of innate immunity in the brain and they sense pathogens or other inflammatory signals; this property contributes to the activation and assembly of inflammasomes and ultimately leads to caspase-1–induced maturation of the IL-1β cytokine [127,128]. Although a normal level of IL-1β is present in a healthy brain, its overproduction may cause inflammation and the associated pathological complications.

On the other hand, various studies have revealed the overexpression of IL-1β in microglia in the vicinity of Aβ plaques in animal models and AD patients [129,130]. The microglial cells in the CNS phagocytose Aβ plaques; this process initiates lysosomal destabilization and the cytosolic production of cathepsin B, which may act as an endogenous signal and make NLRP3 active [123]. Aβ-mediated NLRP3 activation has been found to upregulate IL-1β, which promotes the formation of microglial cells and the accumulation of neurotoxic inflammatory factors [123,131]. Moreover, NLRP3 triggering plays a substantial role in the pathogenesis of amyloidosis. Similarly, in a transgenic mouse model of AD, an NLRP3 knockout protects from spatial memory dysfunction and reduces the deposition of Aβ [132]. The recruitment of microglia in AD is due to the secretion of neurotoxic components produced by senile neurofibrillary plaques [133]; these cells then phagocytose Aβ deposits and further secrete proinflammatory and chemotactic cytokines to aggravate the neurotoxic effects of Aβ. This observation further supports the functions performed by the activation of endogenous NLRP3 in the brain microglial cells because of formation of a plaque [132]. Therefore, the AD pathogenesis is strongly associated with the microglia-mediated NLRP3 inflammasome activation. Additionally, the restoration of Myb1 (TOM1) also reduces Aβ pathology, thereby highlighting the importance of endosomal adaptors and their associated factors in AD pathogenesis [134]. Depending on AD progression and stage, microglia upon activation adopt the M1 phenotype, and the overexpression of proinflammatory cytokines, such as IL-1β, induces an aberration in microglial cells; this event has a negative impact on the Aβ clearance system [135,136]. Nevertheless, an M2-like phenotype is associated with the production of anti-inflammatory cytokines, which contribute to protection against damage induced by inflammation and promote tissue remodeling [137]. Furthermore, activation of NLRP3 inflammasomes induces the extracellular release of ASC particles that act as DAMPs and consequently attract surrounding macrophages [138]. These micrometer-sized ASC specks have been reported to establish a bond with Aβ and promote plaque formation and misfolded protein accumulation, as observed in the APP/PS1 model of AD [139].

Moreover, the most reliable factor underlying the pathogenesis of AD is chronic localized neuroinflammation. Dysregulation of the interaction pattern between microglia and brain neurons is thought to be a contributing factor of AD pathogenesis [140,141]. The overproduction of cytotoxic molecules and proinflammatory cytokines by brain-resident immune cells, such as microglia, launches signaling pathways in neurons, consequently inducing brain tissue damage [142]. Numerous studies have pointed out that overexpression of IL-1β aggravates AD pathogenesis, owing to tau hyperphosphorylation [143], which inhibits long-term potentiation and affects synaptic plasticity [144,145]. Furthermore, IL-1β inhibition in a mouse model has yielded disease-modifying improvements in the pathology of AD [146]. A recent study suggests that a small-molecule NLRP3 inhibitor (JC-124) exerts a beneficial effect on a mouse model of AD [10], similarly to a knockout mouse model that manifested an improvement in spatial memory [132]. Neuronal damage results in the production of proinflammatory cytokines (IL-1β, TNF-α, and IL-6) and triggers apoptotic mitogen-activated protein kinase p38 (MAPK) [147]. P38 MAPK causes glutamate-mediated neurotoxicity via the N-methyl D-aspartic acid receptor [148,149]. Furthermore, IL-1β production induces nitric oxide synthase (iNOS) surrounding the hippocampus; this induction consequently causes damage and neuronal death [150]. IL-1β expression is significantly associated with NLRP3 inflammasome activation, and pyroptosis induced by the inflammasome is an active area of research. Accordingly, deep insight into the mechanism of NLRP3 inflammasome regulation is urgently needed to further unravel AD pathogenesis.

Moreover, the NLRP3 inflammasome is an attractive drug target in AD and subsequently provides neuroprotection. The development of anti-AD drugs that control the activation of NLRP3 inflammasomes may be a promising way to tackle neuroinflammation. The antagonists for the associated signaling pathways showing the potential to slow down AD progression are listed in Table 1.

The aforementioned studies have provided significant insights into the role NLRP3 inflammasomes play in AD progression; therefore, detailed investigation into the mechanism of inflammasome activation and its contribution to neuroinflammation will be worthwhile. Moreover, Aβ accumulation induces microglial activation via advanced glycation end product (AGE) pathways and TLR [151,152]. All these results support the usefulness of NLRP3 inflammasomes and of their downstream regulators in the identification of protein aggregates or peptides that can drive the pathogenesis of such diseases as systemic amyloidosis, prion diseases, AD, and T2D. Targeting of components of the NLRP3 inflammasome, especially at the initial stages of the disease, may reduce the formation and aggregation of Aβ plaques to delay neurological damage in these patients.

## 4. AI-Based Interventions in Advanced Therapeutics

The complexity of a biological system in the normal physiological condition as well as in the development of disease can now be systematically explored, and the relevant data can be mined at a faster rate using cutting edge technologies. Moreover, therapeutic development pipelines, which have been expensive, time-consuming, and complex processes, are being revolutionized by computational and high-throughput techniques. The emergence of high-throughput techniques with applications to biology and medicine gives the pharmaceutical and biotechnology industries unprecedented opportunities and challenges. In the present era, the process of drug development is increasingly dependent on the omics technology owing to its substantial role in the investigation of disease etiology, in identification of druggable targets, and in quality control of drugs throughout their life cycle [153].

Drug discovery is a complex and lengthy process which is composed of four broad stages: (a) selection and validation of target; (b) screening of compound and its lead optimization; (c) preclinical studies; and (d) clinical trials. The first step involves the identification of a disease-associated target which is done by bioinformatics predictions, proteomic and genomic analysis, and genetic and cellular target evaluation. In the next step, target-specific hits are identified from molecular libraries by applying methods such as virtual screening, high-throughput screening, and combinatorial chemistry. The functional properties of drug candidates can be improved by combining in silico and structure–activity studies with cell-based functional tests. Later, in vivo analyses such as toxicity and pharmacokinetics are performed in various animal models. Finally, compounds successfully passing preclinical stages get entered into clinical trials on patients. The clinical evaluation consists of three phases: Phase I involves testing of drug safety on a small number of humans, Phase II involves testing of drug efficacy on a small number of patients with associated disease, and Phase III involves testing drug efficacy on a large number of patients. After confirming the safety and efficacy, compounds are approved and commercialized by the agencies such as FDA. The estimated cost of traditional drug discovery is around 2.6 billion USD with a timeline over 12 years. The huge cost and timeline are the main concerns for most of the pharmaceutical companies. These days, it is becoming possible to improve cost and time by implementing AI-based methods at different stages of drug design and development. For example, AI is being used in cell classification [154], cell sorting [155], calculating compound properties [156], designing new drug-like molecules [157], computer-aided synthesis of compounds [158,159], predicting the 3D structure of targets, and assay development [160,161,162,163]. These processes are hard to perform but can be optimized and automated by implementing the AI-approaches which could significantly speed up the drug discovery process.

**Figure 2:**
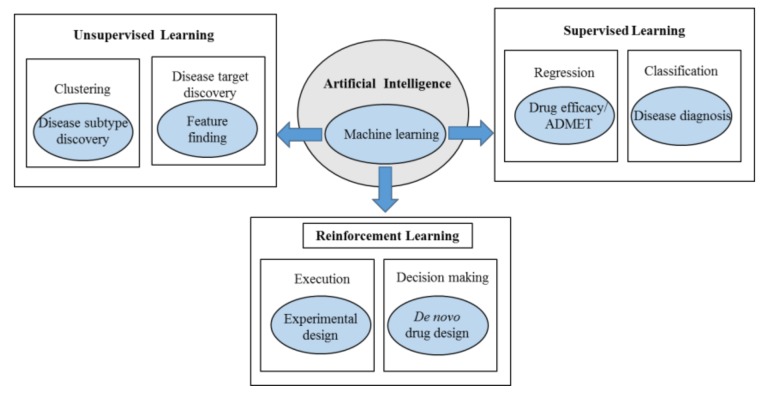
The applications of artificial intelligence (AI) and its subset (machine learning) in disease diagnosis and drug development.

AI includes computational methodologies that simulate or mimic the procedures supported by human intelligence including sensory understanding, learning, reasoning, interaction, and adaptation. AI implications can be found in various fields like image and voice recognition, natural language processing, expert systems, and robotics. Rapidly growing and dynamic methods of AI ensure its applications in the field of medicine as well (Figure 2) [164]. The successful entry of AI into the field of drug discovery has received much attention due to the penetration of AI into all stages of R&D related to drug discovery. AI has helped to discover novel biologically active molecules through ML and deep learning (DL) algorithms on the previously generated quantitative structure–activity relationships (QSARs) and quantitative structure–property relationships (QSPRs) [165].

Investigation of early-phase drug discovery and development has been focused on two levels: activity prediction and classification of molecules. For activity prediction, ML methods including the support vector machine (SVM) algorithm, random forest method, and Bayesian neural networks (BNN) have been employed in the analysis of biological activity of molecules. As far as classification of molecules is concerned, logistic regression, neural networks (NN), and SVM algorithms have been applied to druglike and nondruglike classification. Naïve Bayes (NB) and k-nearest neighbor (KNN) algorithms have also been used to distinguish active molecules from inactive ones. SVM and naïve Bayesian classifications have been successfully employed to categorize lymphocyte-specific protein tyrosine kinase, cytochrome P450, and butyrylcholinesterase inhibitors [166]. Further studies also confirmed the use of SVM-based algorithms to rank molecules based on their activity, whereas BNN and RF are also utilized for activity prediction [167,168,169,170]. More recently a virtual screening was performed to identify cyclin-dependent kinase 2 (CDK2) inhibitors by utilizing protein–ligand interaction fingerprints (PLIF), docking, and SVM. This multistage virtual screening has led to the identification of two novel CDK2 inhibitors that successfully displayed selective inhibition against CDK2 [171]. Table 2 shows programs using ML techniques.

For VS comparison studies, SVM is extremely reliable [181,182] and has been found to identify novel compounds. This algorithm has been successfully employed to find PTP1B inhibitors for the effective treatment of T2D. In this context, five compounds have been identified, out of which two have been experimentally confirmed to have a better inhibitory effect [182]. Moreover, SVM-based computational workflow was utilized for molecular docking calculations to investigate novel inhibitors of c-Met tyrosine kinase.

However, the evaluation of various virtual screening methods is significantly important to confirm their reliability and output. The quality of the evaluation is directly associated with the quality and availability of benchmarking datasets. The successful utilization of benchmarking datasets to optimize a virtual screening methodology has led to the identification of novel compounds. In this context, several benchmarking datasets are available. The DUD-E database is composed of a large number of datasets with various targets such as ion channels and GPCRs (102 protein targets) with clustered ligands (22886) extracted from ChEMBL [183]. The DUD-E database was designed to address some shortcomings of the original DUD version which are particularly related to the weakness of ligand and decoy as charge was not included at the time of property-matching selection of the decoys. Similarly, the DEKOIS 2.0 library is composed of novel 81 structurally distinct benchmark datasets for a huge variety of target classes which could be used for the identification of both decoys and active compounds. This database also contains the information on non-classical binding sites i.e., allosteric binding sites or target protein–protein interaction [184]. Nuclear receptors (NRs) belong to an important class of proteins for drug-targeting so their associated ligands and structures are being evaluated by the Nuclear Receptors Ligands and Structures Benchmarking Database (NRLiSt BDB) [185]. This database contains both agonists and antagonists for 27 human NRs based on experimentally-resolved ligands and target structures which assist in assessing the performance of virtual screening techniques for NRs. Histone deacetylases (HDACs) are an important class of drug targets for the treatment of neurodegenerative diseases, cancers, and other types of diseases. The maximal unbiased benchmarking datasets were constructed to facilitate the discovery of their associated inhibitors. The relevant database is composed of 14 datasets: five of them (HDAC5, HDAC6, HDAC9, HDAC10, and HDAC11) were used to assess ligand-based virtual screening as no experimentally-resolved structures are available for these protein targets [186,187]. More recently, the DeepScreening web server, designed with an integration of deep learning-based algorithms, uses either the user-provided or publically available datasets to assist virtual screening of drugs against the targets of interest [173].

Recently, DL approaches were developed during research on artificial neurons; the purpose was to design the algorithms that automatically discover convoluted patterns and high-level features in large datasets. Such ML algorithms can deal with a diverse and large dataset and uncover complicated relations in the data [188]. Moreover, DL includes a class of ML algorithms that are skilled enough to combine raw inputs into layers of intermediate attributes. Such DL techniques can really help to decipher problems associated with data-rich disciplines, as automated algorithms mine a significant pattern that could lead to actionable knowledge and change how we study diseases, categorize patients, and treat diseases [165]. The development of therapeutics for polygenic and multifactorial diseases is a cumbersome process. In this context, the polypharmacology of compounds seems promising for treating multifactorial diseases such as T2D and AD because it enables a single drug to inhibit multiple targets; this situation may increase its effectiveness against diseases. This idea has led to the development of approaches to the screening of multitargeted directed ligands (MTDLs) [189]. Recent studies utilized VS for AD drug screening for MTDLs that are intended to inhibit multiple associated targets. In this regard, two significant targets—GSK-3β and CDK5—were inhibited by means of sequential docking to screen compounds, whereas ligand-based virtual screening was performed in another study on inhibition of GSK-3β and BACE1 [190,191]. Nonetheless, recursive partitioning (RP) and NB, ML-based VS methods were used to screen compounds for MTDLs against AD. Screening against 25 targets including GSK-3β, BACE1, CDK5, APP, and mAChR (M1 subtype) was performed to search for the compounds that show bonding with a maximum number of targets. Recently available AD-related drugs have been used to further validate this model.

ANN has emerged as an efficient algorithm, and there is a high-performance technique for ANN-based VS against various diseases such as AD and T2D. The ease with which multitask learning (MTL) can be implemented by ANN makes it suitable for screening of candidate compounds for MTDLs. Moreover, the convolutional neural network (CNN) and its application in QSAR modeling such as AtomNet™ make the prediction of small molecule binding to protein targets more promising. Here we propose a more efficient way to perform VS: by means of deep CNN against MTDLs that inhibit multiple drug targets associated with the aforementioned diseases. In case of MTDLs, it is important not to include those targets that are critical for normal physiological functioning and to not interfere with critical pathways ensuring normal homeostasis. Furthermore, the data should be retrieved from the chemogenomic libraries that serve as a training dataset and should include many known inhibitors against the target of interest. These data should be preprocessed as per the standard procedures prior to the use of these data to train and validate CNN internally. The selection of a specific CNN architecture is an area of further research and cannot be suggested here.

Accordingly, if the model is accurately constructed, it can then be implemented on large datasets that are composed of compounds different from the molecules present in the initial training and data testing. The molecules from the top hits should next be tested experimentally to measure the accuracy and efficacy of each selected compound. In conclusion, extensive research activities are underway to integrate AI tools in order to expedite the drug discovery process, but more research is necessary to achieve the full potential of AI in the context of drug discovery and development.

## 5. Concluding Remarks

The proper activation of the immune system is beneficial for the human body in order to combat various immune, inflammatory, degenerative, and metabolic diseases. A slight imbalance in a system could induce various metabolic variations at a cellular or organismic level. These variations activate immune cells which consequently switch on the NLRP3 inflammasome. Being a significant component of the innate immune system, this inflammasome plays a crucial part in metabolic and neurodegenerative disorders such as T2D and AD, respectively. Regardless of the complexity of the pathway, some progress has been made in the development of therapeutics that target the NLRP3 inflammasome and its associated pathways. Nevertheless, further insight is necessary to fully elucidate the pathways that will increase our understanding how to locate precise drug targets and which ones may lead to better treatments. Currently, a variety of inhibitors are available that target activation of the NLRP3 inflammasome and that have been proven to be effective in relieving the symptoms of T2D and AD in patients or in animal models. On the other hand, an advanced therapeutic intervention into such multifactorial diseases is needed to obtain better therapeutic results. In this regard, the research on a cutting edge technology such as AI and ML is laying the foundation for the development of therapeutics with greater efficacy. Moreover, such multifactorial diseases as T2D and AD may be treated effectively by means of VS with deep CNN against MTDLs.

## Figures and Tables

**Table 1:** NLRP3 antagonists in type 2 diabetes mellitus (T2D) and Alzheimer’s disease (AD).

Disease	Target	Intervention/treatment	References
Type 2 diabetes	Interleukin 1 receptor antagonist (IL-1Ra)	Anakinra	[106]
	Anti-interleukin-1β (IL-1β) antibody	Canakinumab	[107]
	NLRP3 (inhibition)	Isoliquiritigenin	[108]
	NLRP3 (inhibition)	Apelin	[109]
	NLRP3 (inhibition)	Sodium butyrate	[110]
	NLRP3 (inhibition)	Glyburide	[88]
	NLRP3 (reduced activation)	Dapagliflozin (Na+ glucose cotransporter 2 inhibitor)	[111]
	NLRP3 (reduced activation)	Empagliflozin	[112]
Alzheimer’s disease	NLRP3 (inhibition)	JC-124	[10]
	NLRP3 (inhibition)	MCC950	[113]
	NLRP3 (inhibition)	β-Hydroxybutyrate (BHB)	[114]
	NLRP3 (inhibition)	Edaravone	[115]
	Aβ1-42–NF-κB pathway (inhibition)	Oridonin	[116]
	NF-κB (inhibition)	TO901317 (LXR agonist)	[117]
	NLRP3 (inhibition)	CY-09	[118]

**Table 2:** Algorithms/programs using machine learning (ML) techniques.

Program	Model/algorithm	Input features	Application	References
AtomNet	DCNN	Molecular graph	Bioactivity prediction of small molecules	[172]
DeepScreening	DNN	Molecular fingerprints	Virtual screening web server	[173]
MLViS	SVM	Physicochemical features (logP, PSA, DC, AlRC, ArRC and BI)	Classify molecules as drug-like and nondrug-like	[166]
MoDeSuS	LR, RT, NN, kNN, RF	Molecular descriptors	Selection of molecular descriptors	[174]
DPubChem	RF, SVM, NB, SVM, KNN	Topological finger prints and chemical descriptors	QSAR modeling and high-throughput virtual screening	[175]
AutoQSAR	MLR, PLS, PCR, NB, RP	Descriptors and fingerprints	Validate and deploy QSAR models.	[176]
SitePredict	RF	Residue-based site properties including spatial clustering of residue types and evolutionary conservation	Prediction of binding sites (small molecules, metal ions)	[177]
DoGSiteScore	SVM	Physicochemical properties	Pocket and druggability prediction	[178]
SCREEN	RF	Physicochemical, structural, and geometric attributes.	Pocket prediction and characterization	[179]
Nnscore 2.0	NN	Receptor–ligand scoring function	Identification of small-molecule ligands	[180]

DCNN: deep convolutional neural network, DNN: deep neural network, SVM: support vector machine, LR: linear regression, RT: regression trees, NN: neural networks, KNN: k-nearest neighbors, RF: random forest, MLR: multiple linear regression, PLS: partial least square regression, PCR: principal components regression, RP: recursive patriating, PSA: polar surface area, DC: donor count, AlRC: aliphatic ring count, ArRC: aromatic ring count, BI: balaban index.
